# Fully‐Conjugated Covalent Organic Frameworks with Two Metal Sites for Oxygen Electrocatalysis and Zn–Air Battery

**DOI:** 10.1002/advs.202206165

**Published:** 2023-01-22

**Authors:** Jiawen Li, Peng Liu, Jianyue Yan, Hao Huang, Wenbo Song

**Affiliations:** ^1^ College of Chemistry Jilin University Changchun 130012 P. R. China; ^2^ Department of Microsystems University of South‐Eastern Norway Borre 3184 Norway

**Keywords:** bimetal sites, covalent organic frameworks, electrocatalysis, fully‐conjugated structure, oxygen reduction reaction, Zn–air battery

## Abstract

Covalent organic frameworks (COFs) are a promising alternative toward catalysis, due to the unique framework structure and the excellent chemical stability. However, the scarcity of unsaturated metal sites and the low conductivity have constrained the advancement of these materials for catalysis of electrochemical reactions. Exploring next‐generation conductive metal–covalent organic frameworks (M‐COFs) with extra metal active sites is crucial for improving their catalytic activity. Herein, a novel fully‐conjugated M‐COFs (Co‐PorBpy‐Co) with two types of metal sites is proposed and achieved by solvothermal method in the presence of carbon nanotube (CNT). The electrocatalyst constructed by the Co‐PorBpy‐Co exhibits excellent oxygen reduction reaction (ORR) activity (*E*_1/2_ = 0.84 V vs RHE, *n* = 3.86), superior to most COFs‐based catalysts. Theoretical result shows the Co—N_2_ sites are extremely active for ORR, and Co‐PorBpy‐Co exhibits excellent conductivity for electron transfer. The Zn–air battery constructed by Co‐PorBpy‐Co/CNT manifests excellent power density (159.4 mW cm^−2^) and great cycling stability, surpassing that of 20 wt% Pt/C catalyst. This work not only proposes a novel design concept for electrocatalysts, but establishes a mechanism platform for single‐metal atom electrocatalysis and synergistic effect.

## Graphical abstract

A novel porphyrin‐based covalent organic frameworks is developed by engineering two single‐metal sites (Co—N_2_ and Co—N_4_) in one framework. Due to the multiple structural advantages and high metal content (≈10.25 wt%), the electrocatalyst constructed from Co‐PorBpy‐Co exhibits excellent oxygen reduction reaction activity (*E*_1/2_ = 0.84 V vs RHE, *n* = 3.86), outperforming nearly all COF‐based catalysts.

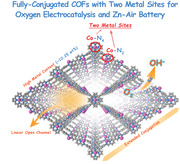

## 1 Introduction

In the post‐oil era, metal–air batteries are fast becoming an essential technology for clean energy utilization.^[^ ^1^, ^2^, ^3^ ^]^ Owing to the sluggish kinetics, the oxygen reduction reaction (ORR) severely delays the efficiency of metal–air batteries.^[^ ^4^, ^5^, ^6^, ^7^ ^]^ Nowadays, many noble‐, transition‐metal inorganic nanostructures were exploited as electrocatalysts for ORR. However, the electrocatalytic activities of most inorganic catalysts arises from the unsaturated metal sites exposed on the surfaces or edges, and the internal metal atoms in bulk phase are totally inert, resulting in the low utilization and efficiency of metal atoms for ORR electrocatalysis.^[^ ^8^ ^]^ Exploring novel electrocatalysts with high metal utilization is essential for the development of metal–air batteries.^[^ ^9^ ^]^

In the past decade, single‐atom catalysts (SACs)^[^ ^10^, ^11^, ^12^ ^]^ strategies have been proposed to promote the efficiency and utilization of metal atoms in electrocatalysts. In particular, atomic‐level M–N*_x_* sites not only offer extremely high metal atom utilization, but also exhibit competitive ORR activity with noble metal catalysts.^[^ ^10^ ^]^ Since the preparation of atomic M–N*_x_* sites relies on high‐temperature pyrolysis, the differences in precursors and temperature can lead to significant discrepancies in their electrocatalytic activities.^[^ ^13^ ^]^ Thus, the repeatability of the single‐atom catalysts is a new raising challenge for the study of structure–activity relationship.^[^ ^14^ ^]^ Meanwhile, as the result of the metal agglomeration in the pyrolysis process, the metal content is still at a very low level (≤1 at%),^[^ ^10^, ^15^ ^]^ which extremely obstructs the properties of SACs. In this regard, exploiting pyrolysis‐free ORR electrocatalysts with well‐defined structures and controllable single metal sites is crucial for the development of SACs.^[^ ^16^, ^17^, ^18^ ^]^

Inspired by the single atomic metal sites and uniform porous structures, metal–organic frameworks (MOFs)^[^ ^19^ ^]^ can be emerged as efficient catalysts, but the insufficient chemical stability of MOFs materials greatly obstructs their widespread applications in electrocatalysis.^[^ ^20^ ^]^ On the other hand, covalent organic frameworks (COFs)^[^ ^21^ ^]^ possess an outstanding stable framework, constructed by covalent bonds,^[^ ^22^, ^23^, ^24^, ^25^, ^26^, ^27^ ^]^ but the non‐metal frameworks also limit their electrocatalytic activity due to the lack of metal sites in the meantime. In order to break through this bottleneck, the metal–covalent organic frameworks (M‐COFs),^[^ ^28^ ^]^ constituted by the metal coordinated structure of MOFs and the covalent framework of COFs, have been considered as the “bridge” between MOFs and COFs, which should be a promising alternative for electrocatalysis.

**Figure 1:**
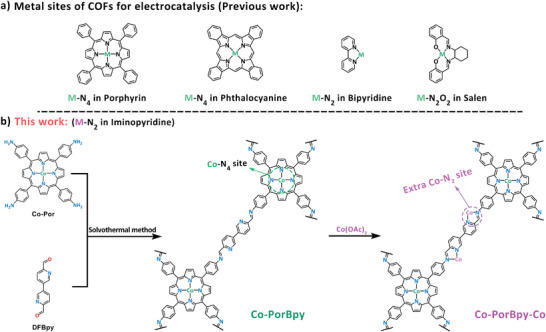
a) The typical metal sites species for M‐COFs, b) the engineering method for two metal sites in M‐COFs.

Similar to SACs, the metal sites in M‐COFs are existed as single‐metal sites, offering excellent catalytic activities toward electrocatalysis.^[^ ^23^ ^]^ Recently, few M‐COFs have been reported as catalysts for various electrochemical reactions (**Figure** **1**a), such as porphyrin‐,^[^ ^29^, ^30^ ^]^ bipyridine‐,^[^ ^31^, ^32^ ^]^ and salen‐based M‐COFs.^[^ ^33^, ^34^ ^]^ However, the relatively low metal content and scanty metal site species in the existing M‐COFs present a new challenge for further increasing the electrocatalytic activity. As far as known, the metal porphyrin‐based M‐COFs usually possess an excellent *π*‐conjugated structure, enabling the fast charge transfer in electrocatalytic reactions.^[^ ^35^ ^]^ The metal sites in bipyridine‐based M‐COFs have highly unsaturation (M—N_2_ moieties) and manifest a superior intrinsic activity.^[^ ^32^ ^]^ Therefore, predictably, the combination between porphyrin and bipyridine subgroups could significantly increase the electrocatalytic activity by four advantages: 1) the bimetal active sites with high metal content and unsaturation; 2) two types of metal sites (M–N_2_ and M–N_4_) for synergistic catalysis, 3) the fully *π*‐conjugated structure with excellent conductivity for electron transfer, and 4) the expanded the porous structure for fast mass transfer. In this respect, the as‐designed M‐COFs with porphyrin‐bipyridine moieties enable a diversity of modulation strategies, including metal modulation and dual‐metal sites interaction, and can be served as an excellent platform for the research on single‐metal electrocatalytic mechanisms, which will make an important contribution to the field of electrocatalysis. Recently, some literature has reported the CNT hybrid catalysts,^[^ ^18^, ^36^ ^]^ revealing the critical role of CNT in charge transfer enhancement. Inspired by these reports, a CNT incorporation strategy could be another effective way to enhance the ORR electrocatalytic activity as well as Zn–air battery performance.

Herein, a new porphyrin‐bipyridine‐based COFs with two metal sites (Co‐PorBpy‐Co) was designed and synthesized by using 5,10,15,20‐tetra(4‐aminophenyl)‐21H,23H‐porphyrin cobalt (Co‐Por with Co—N_4_) and 3,3″‐bipyridine‐6,6″‐dicarbaldehyde (DFBpy) via a facile solvothermal method. Importantly, the condensation between the DFBpy and amino groups in Co‐Por can introduce abundant bidentate nitrogen sites for coordinating extra Co atoms (M—N_2_), exhibiting two times metal sites than that of traditional 2,2′‐bipyridine moiety. Density functional theory (DFT) calculations unveil that Co single‐metal sites, especially Co—N_2_ sites, possess an outstanding intrinsic activity and selectivity for ORR, meanwhile the as‐designed Co‐PorBpy‐Co slab manifests a very narrow band gap for excellent conductivity. Moreover, we adopted the CNT incorporation strategy to enhance the charge transfer ability. As the result, Co‐PorBpy‐Co/CNT displays an excellent ORR electrocatalytic activity (*E*_1/2_ = 0.84 V vs RHE, *n* = 3.86), better than most COFs‐based catalysts. The Zn–air battery fabricated by Co‐PorBpy‐Co/CNT has a great peak power density (159.4 mW cm^−2^) with favorable cycling stability. Our work proposes a novel design concept and mechanism platform for electrocatalysts, sheds light on the exploration of M‐COFs, and broadens their application fields.

## 2 Results and Discussion

### 2.1 Synthesis and Characterization

**Figure 2:**
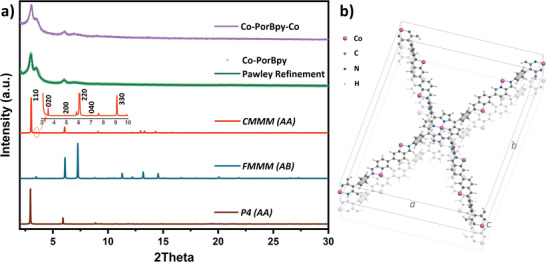
a) The simulated and experimental PXRD patterns of Co‐PorBpy and Co‐PorBpy‐Co, b) the cell model of Co‐PorBpy‐Co.

In this work, Co‐PorBpy frameworks were synthesized by using Co‐Por and DFBpy ligands via a solvothermal method (Figure 1b). First, powder X‐ray diffraction (PXRD) technology was utilized to confirm the crystallization of Co‐PorBpy frameworks. In accordance with the symmetry of Co‐Por (*C*_4_) and DFBpy (*C*_2_), three presumable COFs structures were simulated, defined as CMMM, FMMM, and P4, and their chemical structures are shown in Figure S1, Supporting Information. As shown in **Figure** **2**a, the simulated PXRD pattern of CMMM agrees well with the experimental pattern of Co‐PorBpy framework. The diffraction peaks at 3.04°, 3.50°, 6.09°, and 6.99° can be allocated to (110), (020), (220), and (040) facets, respectively. Moreover, Pawley‐refinement was also utilized to obtain the precise cell parameters of Co‐PorBpy. Pawley‐refined model, simulated by using Cmmm space group with lattice parameters (*a* = 35.7976 Å, *b* = 50.1668 Å, *c* = 6.8319 Å, *α* = *β* = *γ* = 90°), is in good agreement with the experimental data (*R*_wp_ = 5.40%, *R*_p_ = 4.19%), indicating the achievement of Co‐PorBpy frameworks. The atomic coordinates of Co‐PorBpy cell have been provided in Table S1, Supporting Information.

In the Co‐PorBpy frameworks, the imine‐pyridine moieties are formed by the condensation between aldehyde groups in DFBpy and imine groups in Co‐Por, which offer two coordinated sites to introduce extra metal atoms as M—N_2_ sites.^[^ ^37^ ^]^ After a facile coordination reaction, the Co‐PorBpy‐Co frameworks with extra Co—N_2_ sites were obtained (Figure 2b). The PXRD pattern of Co‐PorBpy‐Co is similar to that of Co‐PorBpy sample, indicating the structure and crystallization of COF still keep the crystal structure after the coordination reaction.

**Figure 3:**
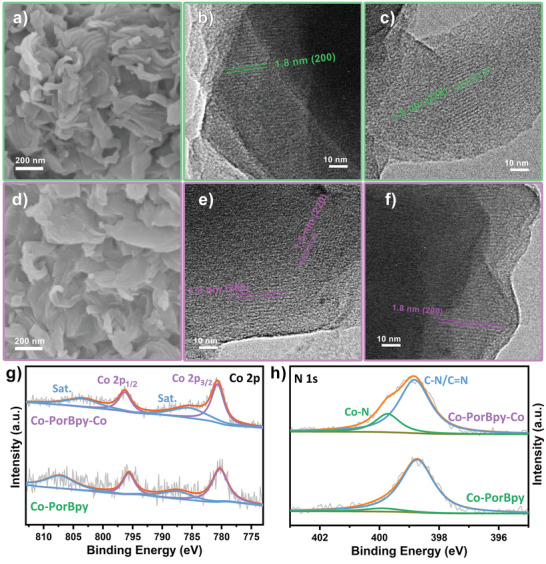
The a) SEM and b,c) TEM images of Co‐PorBpy, d) SEM and e,f) TEM images of Co‐PorBpy‐Co, g) Co 2p and h) N 1s XPS spectra of COFs.

To inspect the morphology of these frameworks, scanning electron microscopy (SEM) was undertaken in the meantime. The SEM images reveal the Co‐PorBpy is composed of stacked leaf‐like sheets (**Figure** **3**a). The SEM images of Co‐PorBpy‐Co show similar morphology and size with Co‐PorBpy (Figure 3d; Figure S6, Supporting Information). The transmission electron microscopy (TEM) images show the stacked nanosheets in these MCOFs (Figure 3b,c,e,f). Moreover, high‐resolution TEM images manifest the 1.8 and 1.5 nm spacings, which belong to the (200) and (220) facets in Co‐PorBpy and Co‐PorBpy‐Co frameworks.

X‐ray photoelectron spectroscopy (XPS) was adopted to inspect the surface chemical states. The XPS results demonstrate the existence of Co, C, N, and O elements in Co‐PorBpy‐Co frameworks, where the oxygen element originates from the absorbed water molecules on the surface of the M‐COFs (Figure S2, Supporting Information). In the Co 2p spectrum (Figure 3g), there are two peaks (795.7 and 780.3 eV) can be allocated to Co 2p_1/2_ and Co 2p_3/2_ peaks of Co‐PorBpy, indicating the presence of Co (II). Compared with the Co‐PorBpy sample, the Co 2p peak of Co‐PorBpy‐Co shifts to a higher energy (796.4 and 780.7 eV) due to the introduction of Co—N_2_ site. The peak shift indicates that the Co—N_2_ sites can effectively adjust the electronic structure of overall Co sites in the MCOFs, which could affect the electrocatalytic performance of the Co‐PorBpy‐Co. The N 1s spectrum shows two subpeaks at 398.8 and 399.9 eV, separately belonging to the chemical bonds of C—N/C=N and Co—N species (Figure 3h). Particularly, the ratio of Co—N species in Co‐PorBpy‐Co is much higher than that in Co‐PorBpy sample, which directly confirms the introduction of Co—N_2_ site as well as the result of Co XPS. For the C 1s spectrum (Figure S3, Supporting Information), four obvious subpeaks can belong to the C=C/C—C (284.6 eV), C—N (285.2 eV), C=N (285.8 eV), and C=O (289.3 eV for Co‐PorBpy; 288.0 eV for Co‐PorBpy‐Co). Noteworthy, the C=O peak in Co‐PorBpy sample appears higher than that of Co‐PorBpy‐Co. The surface element content was also calculated by XPS results. As shown in Table S2, Supporting Information, the Co content in Co‐PorBpy is about 0.90 at%, is much lower than that of Co‐PorBpy‐Co (2.27 at%), suggesting the existence of Co—N_2_ moieties in the Co‐PorBpy‐Co. Notably, the Co‐PorBpy‐Co exhibits an ultra‐high Co content (10.25 wt%) that exceeds most M‐COFs and SACs. Moreover, the thermogravimetric analysis (TGA) was conducted to further confirm the Co content of Co‐PorBpy‐Co. As shown in Figure S5, Supporting Information, the mass of Co‐PorBpy‐Co greatly decreased at ≈350 °C, which was due to the framework decomposition and cobalt oxide formation.^[^ ^38^ ^]^ The Co content was calculated to be 13.68 wt%, higher than the content measured by XPS, confirming the extremely high metal content of Co‐PorBpy‐Co.

To prove the universality of M—N_2_ site introduction strategy, we synthesized Co‐PorBpy‐Ni and conduct XPS analysis. As shown in Figure S4a, Supporting Information, Co 2p spectra also show the existence of Co(II) in Co‐PorBpy‐Ni. Importantly, N 1s spectra can be deconvoluted into three subpeaks, belonging to the C—N/C=N (398.8 eV), Ni—N (399.7 eV), and Co—N (399.9 eV), indicating the successful introduction of extra Ni—N_2_ sites (Figure S4b, Supporting Information). The C1s spectrum of Co‐PorBpy‐Ni shows four subpeaks similar to Co‐PorBpy‐Co (Figure S4c, Supporting Information) and the Ni 2p spectrum indicates the presence of Ni(II) (Figure S4d, Supporting Information). Moreover, the metal content was also measured by XPS. As shown in Table S2, Supporting Information, Ni content of Co‐PorBpy‐Ni is 1.26 at%, higher than the Co content (1.11 at%), showing the validity of M—N_2_ site introduction in enhancing the metal content of M‐COFs.

**Figure 4:**
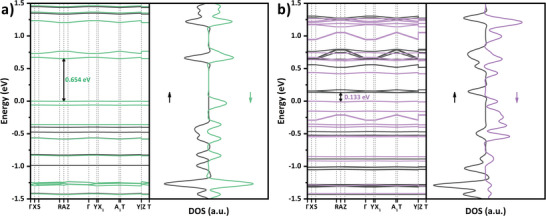
The band structures and DOS of a) Co‐PorBpy and b) Co‐PorBpy‐Co.

The electronic structure can reflect the charge transport properties of electrocatalysts, which can indirectly affect their catalytic performance.^[^ ^30^, ^39^ ^]^ DFT calculations were conducted to reveal the band structure and density of states (DOS) of M‐COFs. According to the Cmmm space group of M‐COFs, the primitive cell of C‐centered orthorhombic (ORCC) lattice was employed for the DFT calculations (Figure S7, Supporting Information).^[^ ^40^ ^]^ Band structure calculations demonstrate that both Co‐PorBpy and Co‐PorBpy‐Co are semiconductors (**Figure** **4**). The Co‐PorBpy exhibits a band gap of 0.654 eV with flat bands, due to the localization of electron density.^[^ ^41^ ^]^ On the other hand, the Co‐PorBpy‐Co shows a narrower band gap of 0.133 eV, indicating a more efficient in‐plane charge transport rather than Co‐PorBpy. Above results indicate that the introduction of Co—N_2_ sites greatly promotes the charge transfer efficiency, which is beneficial to improving the electrocatalytic ORR activity of Co‐PorBpy‐Co.

### 2.2 Electrocatalytic Performance of M‐COFs

**Figure 5:**
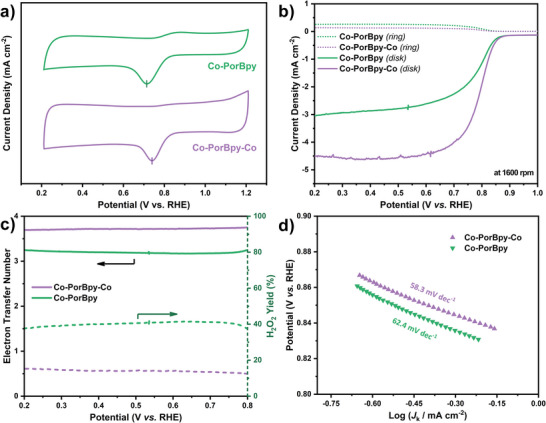
a) The CV curves, b) LSV curves at 1600 rpm, c) electron transfer number and hydroperoxide yield plots, and d) Tafel plots of Co‐PorBpy and Co‐PorBpy‐Co.

The ORR activities of these M‐COFs were conducted in O_2_‐saturated 0.1 m KOH solution. As shown in **Figure** **5**a, cyclic voltammetry (CV) curves were obtained by rotating ring‐disk electrodes (RRDE). There is a cathodic peak of Co‐PorBpy‐Co at 0.74 V (vs RHE), more positive than that of Co‐PorBpy (0.71 V vs RHE), indicating the higher ORR activity of Co‐PorBpy‐Co. Linear sweep voltammetry (LSV) polarization tests were also utilized to estimate the ORR electrocatalytic activities of M‐COFs samples. The half‐wave potential (*E*_1/2_) of Co‐PorBpy‐Co reaches up to 0.79 V versus RHE (Figure 5b), more favorable than that of Co‐PorBpy (0.77 V vs RHE). Thus, the Co‐PorBpy‐Co exhibits a higher *E*_1/2_ (0.79 V vs RHE) and larger *J*_L_ (4.52 mA cm^−2^) than those of Co‐PorBpy sample (0.77 V vs RHE and 3.04 mA cm^−2^), indicating that the presence of Co—N_2_ sites could greatly improve the activity of M‐COFs catalysts.

Selectivity is another vital factor that can reflect the ORR property of electrocatalysts. Both electron transfer number (*n*) and H_2_O_2_ yield were estimated by RRDE analysis (Figure 5c). In this work, the Co‐PorBpy‐Co show good selectivity (*n* = 3.74 at 0.75 V vs RHE) in this ORR process, better than that of Co‐PorBpy (*n* = 3.19 at 0.75 V vs RHE), suggesting the importance of Co—N_2_ sites and CNT substrate on the ORR selectivity. On the other hand, the H_2_O_2_ yield of Co‐PorBpy‐Co is about 13% (at 0.75 V vs RHE), lower than that of Co‐PorBpy (41%). This phenomenon indicates that the introduction of extra Co—N_2_ sites plays a very important role in M‐COF electrocatalysts. Tafel slopes were calculated for these M‐COFs samples to appraise their ORR kinetics. The Tafel slope of Co‐PorBpy‐Co is about 58.3 mV dec^−1^, smaller than that of Co‐PorBpy (62.4 mV dec^−1^), indicating the higher transfer coefficient (*α*) on Co‐PorBpy‐Co (Figure 5d). Importantly, the Tafel slopes of M‐COF catalysts are closed to 60 mV dec^−1^, the rate‐determining step (RDS) in these electrocatalysts may be the first electron transfer process.^[^ ^42^ ^]^

### 2.3 Theoretical Calculations

**Figure 6:**
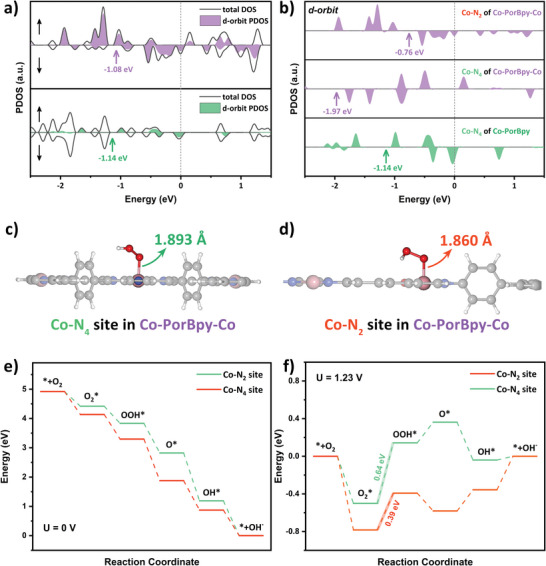
a) The PDOS of Co‐PorBpy and Co‐PorBpy‐Co, b) PDOS of Co metal sites in COFs, binding site illustrations of c) Co—N_4_ and d) Co—N_2_ sites in Co‐PorBpy‐Co, free energy diagrams of Co—N_4_ and Co—N_2_ sites in Co‐PorBpy‐Co at the applied potential of e) 0 V and f) 1.23 V.

DFT calculations were conducted to unveil the function of two‐types Co sites in Co‐PorBpy‐Co. As known, the DOS near the Fermi level (*E*_F_) affects the binding strength between catalyst and adsorbate, which will further affect the catalytic activity.^[^ ^43^ ^]^ The total DOS (TDOS) of COFs near *E*_F_ mainly consists of the d‐orbit projected DOS (PDOS), manifesting the importance of d‐band center (*ε*_d_) (**Figure** **6**a). When *ε*_d_ gets close to the *E*_F_, the interaction between metal sites and intermediates turns stronger, which can enhance the electron transfer and boost the ORR activity.^[^ ^44^ ^]^ The *ε*_d_ of spin‐down d‐orbits was calculated because the spin‐down d‐orbits occupy the higher energy in the spin‐polarized metal sites.^[^ ^45^ ^]^ The *ε*_d_ of Co‐PorBpy‐Co is –1.08 eV, higher than that of Co‐PorBpy (–1.14 eV), showing the improved activity of metal sites in Co‐PorBpy‐Co (Figure 6a). Furthermore, the intrinsic activity of Co—N_2_ and Co—N_4_ sites were revealed by plotting the d‐orbit PDOS of metal sites. The *ε*_d_ of Co—N_2_ in Co‐PorBpy‐Co is −0.76 eV, which is much higher than those of Co—N_4_ in Co‐PorBpy‐Co (−1.97 eV) and Co—N_2_ in Co‐PorBpy (−1.14 eV) and indicates the highest intrinsic ORR activity of Co—N_2_ site. Notably, the *ε*_d_ of Co—N_4_ shifts to lower energy after the introduction of Co—N_2_ sites due to the charge transfer from Co—N_4_ to Co—N_2_ through the conjugated framework.

To further reveal the activity and selectivity, free energy calculations were investigated on the Co—N_2_ and Co—N_4_ sites in Co‐PorBpy‐Co. The M‐COF slabs were built by using a 15 Å vacuum layer to prevent inter‐layer interactions. As shown in Figure 6c,d, the OOH* species are optimized at the Co—N_2_ and Co—N_4_ sites in Co‐PorBpy‐Co slab. The Co—O bond length of Co—N_4_ site (1.893 Å) is longer than that of Co—N_2_ site (1.860 Å), indicating the enhanced bonding strength between Co—N_2_ site and OOH*. Moreover, the free energies were calculated and the diagrams were plotted (Table S3, Supporting Information). All the free energies of Co—N_2_ are lower than those of Co—N_4_ sites, consistent with the results of PDOS analysis (Figure 6e,f). Similar to the result of Tafel slope, the rate‐determining step (RDS) in both Co—N_4_ and Co—N_2_ sites is the step of O_2_*→OOH*. However, the limiting energy barrier of Co—N_2_ is 0.39 eV, much smaller than that of Co—N_4_ site (0.64 eV), which directly indicates that the Co—N_2_ sites in Co‐PorBpy‐Co slab have better ORR activity than Co—N_4_ sites. As an important factor in ORR, selectivity is determined by the energy barrier of OOH*→O*. The energy barrier of OOH*→O* in Co—N_4_ is 0.22 eV, indicating that the desorption of OOH* is thermodynamically favored (the 2e^−^ ORR pathway).^[^ ^46^ ^]^ In contrast, the energy barrier of OOH*→O* in Co—N_2_ is −0.19 eV, indicating the conversion from OOH* to O* is thermodynamically favored as in the 4e^−^ ORR pathway. Based on the above findings, the ORR pathway with elementary steps on Co sites was summarized and drawn in Figure S11, Supporting Information. Overall, the introduction of extra Co—N_2_ sites not only increases the number of metal sites, but significantly enhances the interaction between catalyst and intermediates for the enhanced ORR activity and selectivity of Co‐PorBpy‐Co.

### 2.4 Incorporation Strategy and Zn–Air Battery Tests

**Figure 7:**
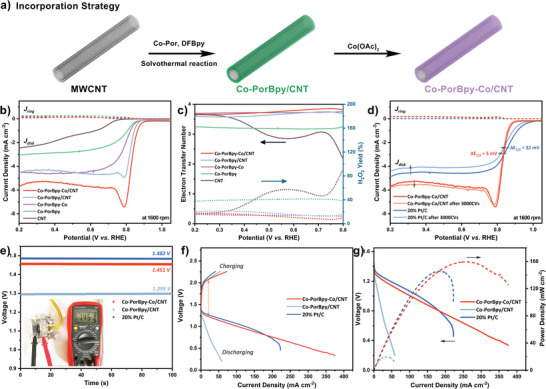
a) The illustration of incorporation strategy. b) The LSVs and c) electron transfer number and hydroperoxide yield plots of the catalysts, d) stability tests of Co‐PorBpy‐Co and 20 wt% Pt/C at 1600 rpm, e) the OCV plots of ZABs with Co‐PorBpy‐Co/CNT, Co‐PorBpy/CNT, and 20 wt% Pt/C, f) charging and discharging polarization curves of ZABs, g) power density curves of ZABs.

For constructing electrocatalysts, many previous works have reported that some carbon substrates, such as carbon nanotubes (CNTs) and graphene not only can enhance the conductivity for faster electron transfer, but orient the growth of COFs for ultrathin layered structure, which brings more exposed metal sites and higher catalytic activity.^[^ ^36^ ^]^ In this work, we incorporated CNTs into the Co‐PorBpy and Co‐PorBpy‐Co by in situ growth for engineering the thin‐layered structure of M‐COFs (**Figure** **7**a). The XRD and TEM results exhibit that the Co‐PorBpy/CNT has a good crystallinity (Figure S8, Supporting Information), and CNTs are essential for the preparation of ultrathin structure on Co‐PorBpy (Figure S9, Supporting Information). The ORR activities of these M‐COFs/CNT were also conducted in O_2_‐saturated 0.1 m KOH solution. As shown in Figure 7b, LSV polarization tests exhibit that the half‐wave potential (*E*_1/2_) of Co‐PorBpy‐Co/CNT reaches up to 0.84 V versus RHE, more favorable than those of Co‐PorBpy/CNT (0.83 V vs RHE), Co‐PorBpy‐Co (0.79 V vs RHE), Co‐PorBpy (0.77 V vs RHE), and CNT (0.56 V vs RHE). Thus, the Co‐PorBpy‐Co/CNT not only possesses the optimal *E*_1/2_, but exhibits the largest diffusion‐limiting current density (*J*_L_) of 5.69 mA cm^−2^, confirming the highest ORR activity of Co‐PorBpy‐Co/CNT. Notably, the ORR activity Co‐PorBpy‐Co/CNT is higher than that of Co‐PorBpy/CNT, indicating that the presence of Co—N_2_ sites could greatly improve the activity of M‐COFs catalysts. Moreover, M‐COF/CNT catalysts exhibit better activity than COFs samples without substrates, suggesting the importance of reduced thickness and increased sites with exposed metal. Moreover, the catalysts with CNT exhibit a kink at 0.78 V versus RHE, which may be due to the removal of adsorbed oxygen molecules during the ORR process.^[^ ^47^ ^]^ Electron transfer number (*n*) and H_2_O_2_ yield were also estimated and analyzed by RRDE analysis (Figure 7c). The *n* of Co‐PorBpy‐Co/CNT reaches 3.86 at 0.75 V versus RHE and slight H_2_O_2_ (only 7%) exists in the electrolyte as a by‐product, which is the best behavior in these M‐COFs‐based samples, implying that water molecules are the ORR product by using Co‐PorBpy‐Co/CNT as catalyst. Further comparison is shown in Figure S10a, Supporting Information, where the ORR activity of Co‐PorBpy‐Co/CNT is optimal, indicating that the introduction of extra Co—N_2_ sites and CNT substrate plays a very important role in M‐COF electrocatalysts.

The Tafel slope of Co‐PorBpy‐Co/CNT is about 51.7 mV dec^−1^, smaller than those of other catalysts, which illustrates the highest transfer coefficient on Co‐PorBpy‐Co/CNT (Figure S10b, Supporting Information). Electrochemical impedance spectroscopy (EIS) tests manifest that Co‐PorBpy‐Co/CNT possesses the smallest *R*_ct_ value (26.12 Ω) among these M‐COFs catalysts, indicating the fast electron transfer between Co‐PorBpy‐Co/CNT and electrolyte during ORR process (Figure S10c, Supporting Information). The ORR activity of commercial 20 wt% Pt/C was investigated for comparison (Figure 7d). The *E*_1/2_ of Pt/C is 0.86 V versus RHE and the *J*_L_ is 4.86 mA cm^−2^. By contrast, the ORR activity of Co‐PorBpy‐Co/CNT is comparable to that of commercial Pt/C catalyst owing to the approximate *E*_1/2_ and higher current density. LSV curves were inspected after the CV cycles for stability tests. After 3000 CV cycles, the E_1/2_ of 20 wt% Pt/C decreases 22 mV, and the *J*_L_ drops by 0.686 mA cm^−2^; in contrast, the *E*_1/2_ of Co‐PorBpy‐Co/CNT only decreases 5 mV, and the *J*_L_ even raises by 0.095 mA cm^−2^. This result illustrates the stability of Co‐PorBpy‐Co/CNT is superior to that of 20 wt% Pt/C in all respects. The ORR activities of some COF‐based catalysts were summarized for comparison (Table S4, Supporting Information). Obviously, the ORR performance of Co‐PorBpy‐Co/CNT is comparable to 20 wt% Pt/C, and superior to most COF‐based catalysts.

Considering the excellent ORR activity of COF/CNT hybrid, a Zn–air battery (ZAB) was assembled with Co‐PorBpy‐Co/CNT as the cathode catalyst and zinc foil as the anode. Co‐PorBpy/CNT and commercial 20 wt% Pt/C were also employed to construct ZAB for comparison. The ZAB with Co‐PorBpy‐Co/CNT manifests a stable open‐circuit voltage (OCV) of 1.451 V (Figure 7e), which is competitive with the ZAB with 20 wt% Pt/C (1.482 V) and higher than that with Co‐PorBpy/CNT (1.295 V). The discharging curve of ZAB with Co‐PorBpy‐Co/CNT is marginally inferior at lower current density but better at higher current density (Figure 7f). Meanwhile, the charging curve of Co‐PorBpy‐Co/CNT is superior to that of Pt/C catalyst, consistent with OER results (Figure S12a, Supporting Information). The voltage gap (Δ*E*) is an important parameter for the performance of ZAB, and a smaller Δ*E* usually exhibits better performance. As the result of the excellent bifunctional oxygen catalytic activity of Co‐PorBpy‐Co/CNT, the Δ*E* between charge and discharge of ZAB with Co‐PorBpy‐Co/CNT is 0.843 V (at 20 mA cm^−2^), smaller than that with 20 wt% Pt/C (0.870 V) and Co‐PorBpy/CNT(1.235 V). As seen in Figure 7g, the power density curves of ZABs were determined from their discharging polarization curves. The ZAB with Co‐PorBpy‐Co/CNT possesses a peak power density of 159.4 mW cm^−2^, surpassing that with 20 wt% Pt/C (145.1 mW cm^−2^). Importantly, the peak power density of ZAB with Co‐PorBpy‐Co/CNT is ≈8 times higher than that with Co‐PorBpy/CNT (19.42 mW cm^−2^), indicating the importance of introducing Co—N_2_ sites for high‐performance. Step discharging curves were utilized to estimate the rate performance of ZABs (Figure S12b, Supporting Information). The discharge voltages of Co‐PorBpy‐Co/CNT cathode are lower than those of Pt/C cathode at current densities less than 20 mA cm^−2^. On the contrary, the discharge voltages of Co‐PorBpy‐Co/CNT are much higher at 50 and 100 mA cm^−2^, indicating that Co‐PorBpy‐Co/CNT has a higher rate performance at high current densities. The cycling performance of ZABs was estimated at 10 mA cm^−2^ (Figure S13, Supporting Information). Obviously, the voltage gap of ZAB with Co‐PorBpy‐Co/CNT is barely changed after 80 cycles with a small round‐trip efficiency^[^ ^46^ ^]^ difference of 2.7% between initial (61.5%) and 80 cycles (58.8%). However, the Δ*E* of ZAB with 20 wt% Pt/C changes significantly during the first 20 cycles, with a large round‐trip efficiency difference of 12.4% between initial (57.9%) and 80 cycles (45.5%), indicating the better cycling stability of Co‐PorBpy‐Co/CNT cathode. Furthermore, the *ΔE* of ZAB with Co‐PorBpy‐Co/CNT after 80 cycles is 0.843 V, much smaller than that with 20 wt% Pt/C (1.248 V). Thus, all results show the ZAB, constructed by Co‐PorBpy‐Co/CNT, has outstanding performance in practical usage.

## 3 Conclusion

A novel Co‐PorBpy‐Co COFs with extra Co—N_2_ sites was proposed and synthesized in this work. The Co‐PorBpy‐Co not only has two‐type metal active sites with high metal content and unsaturation, the synergistic catalysis between two types of metal sites (M—N_2_ and M—N_4_), but possesses fully *π*‐conjugated frameworks and expanded the porous structure for electron and mass transfer. These unique characteristics can make Co‐PorBpy‐Co a promising alternative for ORR electrocatalysis. As the result, the Co‐PorBpy‐Co/CNT displays excellent ORR activity (*E*_1/2_ = 0.84 V vs RHE, *n* = 3.86), outperforming nearly all COF‐based catalysts. DFT calculations unveil the strong interaction between Co—N_2_ sites and oxygen intermediates and Co—N_2_ sites possess better intrinsic activity and selectivity than Co—N_4_ sites Importantly, the rechargeable ZAB, constructed by Co‐PorBpy‐Co/CNT, has an excellent power density (159.4 mW cm^−2^) and cycling stability, surpassing commercial 20 wt% Pt/C. The research of Co‐PorBpy‐Co lays the foundation for the design concept of electrocatalysts, and proposes an ideal mechanism platform for single‐metal atom electrocatalysis and synergistic effect.

## 4 Experimental Section

### Materials

Cobalt (II) acetate tetrahydrate and multi‐walled carbon nanotube (CNT) was purchased from Aladdin. Acetic acid and methanol were purchased from Macklin. 1,2‐Dichlorobenzene and n‐butanol were purchased from Energy Chemical. 5,10,15,20‐tetrakis(4‐aminophenyl)‐21H,23H‐porphyrin (H_2_‐Por) and 3,3″‐bipyridine‐6,6″‐dicarbaldehyde (DFBpy) was purchased from Bide Pharmatech. Other reagents were acquired from Sinopharm Chemical Reagent Company. The above reagents were used without any purification.

### Preparation of Co‐Por

5,10,15,20‐Tetra(4‐aminophenyl)‐21H,23H‐porphyrin cobalt (II) (Co‐Por) was prepared in line with the reported work with a little modification.^[^ ^27^ ^]^ H_2_‐Por (202.4 mg, 0.3 mmol), cobalt acetate tetrahydrate (298.9 mg, 1.2 mmol), methanol (20 mL), *N*,*N′*‐dimethylformamide (30 mL), and chloroform (90 mL) were added to a 250 mL round bottom flask under N_2_. Upon mixture was refluxed (≈80 °C) under N_2_ for 24 h. After reaction, the dark green mixture was transferred to a separatory funnel and washed for three times with water (100 mL). The lower layer was centrifuged and the precipitate was dried overnight in a vacuum oven at 60 °C to obtain Co‐Por (104.7 mg, yield 47.6%) as dark purple solid.

### Preparation of Co‐PorBpy

A 20 mL Pyrex tube was charged with Co‐Por (11.0 mg, 0.015 mmol), DFBpy (5.73 mg, 0.027 mmol), 1,2‐Dichlorobenzene (1.2 mL), and *n*‐butanol (0.8 mL), and aqueous acetic acid solution (6 m, 0.2 mL), followed by 15 min sonication. Above tube was frozen in liquid N_2_ bath and flame sealed under vacuum. After rising to room temperature, the reaction was heated at 120 °C for 3 days. The precipitate was filtered, washed with acetone, then dried overnight under vacuum at 120 °C to gain brown powder.

### Preparation of Co‐PorBpy‐Co

Co‐PorBpy (12.0 mg) was put into a methanol (8.0 mL) solution of cobalt acetate tetrahydrate (30.0 mg, 0.12 mmol) and stirred for 4 h. The precipitate was filtered, washed with plenty of methanol, and then dried overnight under vacuum at 60 °C to obtain black powder.

### Preparation of Co‐PorBpy‐Ni

The preparation of Co‐PorBpy‐Ni was similar to the preparation of Co‐PorBpy‐Co, except that cobalt acetate tetrahydrate was replaced by nickel acetate tetrahydrate (30.0 mg, 0.12 mmol).

### Preparation of Co‐PorBpy/CNT

Co‐PorBpy/CNT was synthesized as the similar procedure of Co‐PorBpy except CNT (16.73 mg) was added into the precursors to achieve the mass ratio of 1:1 (*M*_COF_:*M*_CNT_).

### Preparation of Co‐PorBpy‐Co/CNT

Co‐PorBpy/CNT (12.0 mg) was added to a methanol (8.0 mL) solution of cobalt acetate tetrahydrate (30.0 mg, 0.12 mmol) and stirred for 4 h. The precipitate was filtered, washed with plenty of methanol, and then dried overnight under vacuum at 60 °C to obtain black powder.

### Physical Characterization

PXRD measurements were conducted on a PANalytical B.V. Empyrean having Cu Ka radiation (1.540598 Å) under 40 kV and 40 mA. SEM images were gained on a HITACHI SU8000 microscope. High‐resolution TEM images were obtained on a JEOL JEM‐2200FS microscope. XPS tests were surveyed on an ESCALAB 250 spectrometer using Al Ka excitation. TGA test was conducted using a TA TGA Q500 thermal analyzer system at the heating rate of 5 °C min^−1^ from room temperature to 800 °C in an air atmosphere.

### Electrochemical Measurements

The electrochemical tests were carried out in a three‐electrode cell under CHI 760e electrochemical station at 298 K. The above cell contains RRDE (working electrode), saturated calomel electrode (reference electrode), and graphite rod (counter electrode). 0.1 m KOH aqueous solution was used as the electrolyte. First, catalyst ink was prepared by mixing 5 mg catalysts with 475 µL DMF, 300 µL ultrapure water, 200 µL ethanol, and 25 µL Nafion (0.5 wt% in ethanol). The catalyst ink was well dispersed by ultrasonication for 1 h. Next, 26 µL ink was drop‐casted onto a pre‐polished RRDE (disk area = 0.247 cm^2^) in two times. Lastly, the electrode was left at 298K to dry. CV curves were recorded in N_2_‐ and O_2_‐saturated electrolytes from 0.21 V to 1.21 V (vs RHE) with a scan rate of 0.05 V s^−1^. RRDE tests were performed under WaveVortex 10 Electrode Rotator (Pine Research Instrumentation) with 1600 rpm rotating speed in O_2_‐saturated electrolyte. LSV plots were recorded at a scan rate of 0.01 V s^−1^ with a 95% iR‐correction. EIS measurements were carried out at 0.81 V (vs RHE). The stability test was conducted using 3000 CV cycles at 0.6 V (vs RHE) over 10 000 s. Hydrogen peroxide yield and *n* were obtained from:^[^ ^46^ ^]^

(1) $$n=\frac{4i_{d}}{i_{d}+\frac{i_{r}}{N}}\;$$

(2) $$H_{2}O_{2}\%=\frac{200\frac{i_{r}}{N}}{i_{d}+\frac{i_{r}}{N}}$$

where *i*_d_ and *i*_r_ represent the disk and ring current, *N* represents the collection efficiency (37%). The ring potential was set to constant at 1.2 V versus RHE.

Other experimental procedures, such as Zn–air battery tests and DFT calculation methods are included in the Supporting Information.

## Conflict of Interest

The authors declare no conflict of interest.

## Supporting information

Supporting Information

## Data Availability

The data that support the findings of this study are available from the corresponding author upon reasonable request.
