# Supplementary material for: Fully‐Conjugated Covalent Organic Frameworks with Two Metal Sites for Oxygen Electrocatalysis and Zn–Air Battery
Source: Adv Sci (Weinh). 2023 Jan 22;10(9):2206165. doi: 10.1002/advs.202206165 (PMC10037685; doi:10.1002/advs.202206165)
Supplement: Supplementary file 1 — Supporting Information [file ADVS-10-2206165-s001.pdf]

Supporting Information

Fully-Conjugated Covalent Organic Frameworks with Two Metal Sites for Oxygen Electrocatalysis and Zn-Air Battery

Jiawen Li, Peng Liu, Jianyue Yan, Hao Huang, and Wenbo Song**

J. Li, P. Liu, J. Yan, Prof. W. Song

College of Chemistry, Jilin University, Changchun 130012, P.R. China.

E-mail: wbsong@jlu.edu.cn

Dr. H. Huang

Department of Microsystems, University of South-Eastern Norway, Borre 3184, Norway

E-mail: huanghao881015@163.com

Contents

1. Experimental Procedures 2
2. COF Models 4
3. XPS Spectra 6
4. TGA Test 7
5. SEM Images 7
6. COF Slabs for Band Structure Calculation 8
7. PXRD Pattern 8
8. TEM Images 9
9. ORR Tests 9
10. Theoretical Computation Section 10
11. Electrocatalytic OER and Zn-air Battery Tests 11
12. Comparison Table 12
13. Reference 13

1. Experimental Procedures

Rechargeable Liquid Zn-air Battery Tests: The air electrode utilized in ZAB was constructed by Ni foam, gas diffusion layer, and catalyst layer. The catalyst ink was loaded onto the other side of the carbon paper by drop-casting to obtain the catalyst layer with a loading of 2.0 mg cm^{-2}. The ink was obtained using the same procedure with the electrochemical tests and the real area exposed on the electrolyte is 1 cm^2. A 0.5 mm thick zinc foil was polished and utilized as the anode. The electrolyte of ZAB is 6 M KOH and 0.2 M zinc acetate aqueous solution. All ZAB tests were performed under CHI 760e electrochemical station at room temperature. The pure oxygen flow was used to supply the cathode. The LSVs were measured under a scan rate of 5 mV s^{-1} without iR-correction, then the discharging power density can be calculated as following:[1]

$$\mathrm{P} = U_{dis} \times J_{dis}$$

where P represents the discharge power density, U_{dis} represents discharge voltage, and J_{dis} represents discharge current density. The rate performance of ZAB was estimated by galvanostatic discharge at the current densities of 2, 5, 10, 20, 50, and 100 mA cm^{-2}. Cycling tests were carried out using galvanostatic method for 60 s discharging and 60 s charging at 10 mA cm^{-2}. The round-trip efficiency of ZABs were determined using the following equation:[1]

$$\text{Round} - \text{trip efficiency} = \frac{E_{dis}}{E_{cha}} \times 100\% = \frac{U_{dis}}{U_{cha}} \times 100\%$$

where E_{dis} and E_{cha} were the discharge/charge energy and U_{dis} and U_{cha} were the discharge/charge voltage, respectively.

Theoretical Computations: All the DFT calculations were performed with Quantum ESPRESSO.[2-3] COF models in this work were built using BURAI. PBE under the GGA[4] with PAW[5] pseudopotentials from Pslibrary 1.0.0 was used for SCF calculations.[6] An electronic convergence threshold of 1E-6 eV was set during SCF cycles, and Broyden–Fletcher–Goldfarb–Shanno (BFGS) was chosen for ionic dynamics optimization. A 50 Ry kinetic energy cut-off for wavefunctions and a 500 Ry kinetic energy cut-off for charge density and potential were employed. A mixing mode of "local-TF" was used in electronic step. A starting magnetization of cobalt was set as 0.2 to process the spin-polarized system. A gaussian smearing of 0.01 Ry were set to handle Fermi-surface effects. The Brillouin-zones were set at gamma point for SCF calculations and 2×2×4 for NSCF calculations. DOS and PDOS calculations were performed with DOS and PROJWFC module, respectively. The gaussian smearing of 0.005 Ry was used to plot DOS. Moreover, Fermi level was set as 0 eV before the DOS and PDOS were plotted. The band structure calculations were executed by PW and BANDS modules, using the k-point path of Γ-X-S-R-A-Z-Γ-Y-X_1-A_1-T-Y|Z-T. The reaction free energy is calculated by $\Delta G = \Delta E + \Delta \mathrm{ZPE} - \mathrm{T}\Delta S + \Delta G_U + \Delta G_{pH}$, where ΔE is the total energy difference between reactants and products, ΔZPE is the zero-point energy correction, ΔS is the vibrational entropy change at finite temperature T, $\Delta G_U = -\mathrm{e}U$, where e is the elementary charge, U is the electrode potential, ΔG_{pH} is the correction of the H^+ free energy include from: $\Delta \mathrm{G}_{pH} = kTln10 \times pH$. The free energy corrections of adsorbates were estimated by Atomic Simulation Environment (ASE, a set of tools

and Python modules).[7] The enthalpy and entropy of the ideal gas molecule were taken from the standard thermodynamic Tables.[8] Notably, a vacuum slab of 15 Å was applied along the *c*-axes to reduce the unwanted interactions during the free energy calculations. The d-band center (ε_d) was calculated according to following equation:

$$\varepsilon_{\mathrm{d}} = \frac{\int_{-\infty}^{0} N(\varepsilon)\, \varepsilon \, d\varepsilon}{\int_{-\infty}^{0} N(\varepsilon)\, d\varepsilon}$$

where $N(\varepsilon)$ is the d-orbit DOS, ε is the energy.

2. COF Models

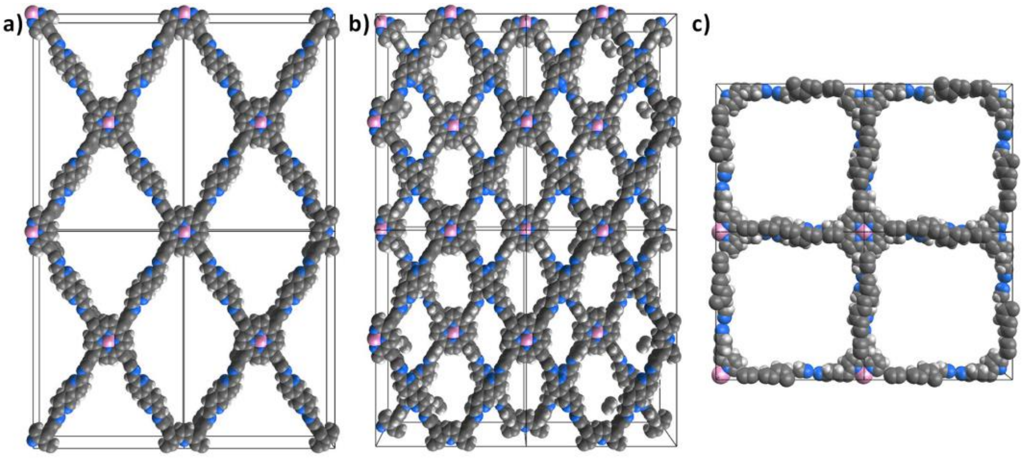

Figure S1. Simulated structures of (a) *CMMM*, (b) *FMMM*, and (c) *P4*.

Table S1. Cell parameters and atomic fractional coordinates of Co-PorBpy model

Space group	*C*mmm		
Cell parameters	a = 35.7976 Å，b = 50.1668 Å，c = 6.8319 Å，α = β = γ = 90°		
Atoms	x	y	z
C1	0.38952	0.41626	-0.17562
C2	0.36682	0.39304	-0.17561
H3	0.39841	0.42441	-0.31442
H4	0.3582	0.38471	-0.31424
C5	0.38331	0.51349	0
C6	0.42045	0.4784	0
C7	0.43097	0.45133	0
C8	0.46944	0.44443	0
C9	0.48092	0.41845	0
C10	0.40139	0.42862	0
C11	0.64463	0.61941	0
N12	0.32905	0.6421	0
C13	0.80293	0.76062	0
C14	0.74939	0.78904	0
N15	0.77314	0.81069	0
C16	0.81159	0.80882	0
C17	0.82657	0.78313	0
C18	0.83865	0.83274	0
C19	0.23689	0.23749	0
H20	0.35855	0.52587	0
H21	0.46357	0.40098	0
H22	0.81659	0.7417	0
H23	0.71954	0.7928	0
H24	0.85677	0.78034	0
H25	0.86843	0.82817	0
N26	0.44405	0.5	0
N27	0.5	0.46102	0
Co28	0.5	0.5	0

3. XPS Spectra

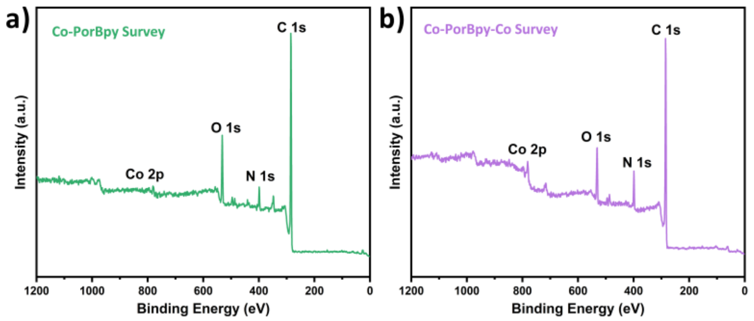

Figure S2. XPS survey spectra of (a) Co-PorBpy, and (b) Co-PorBpy-Co.

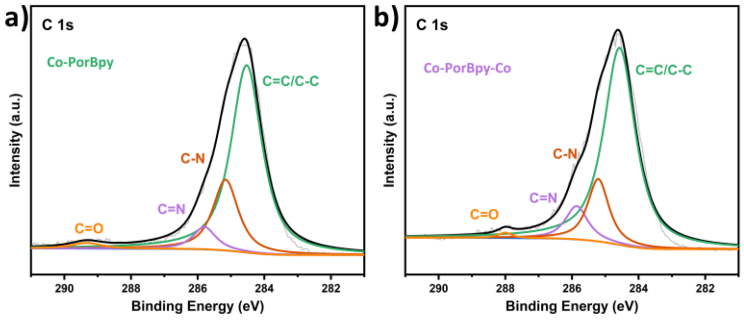

Figure S3. XPS C1s spectra of (a) Co-PorBpy, and (b) Co-PorBpy-Co.

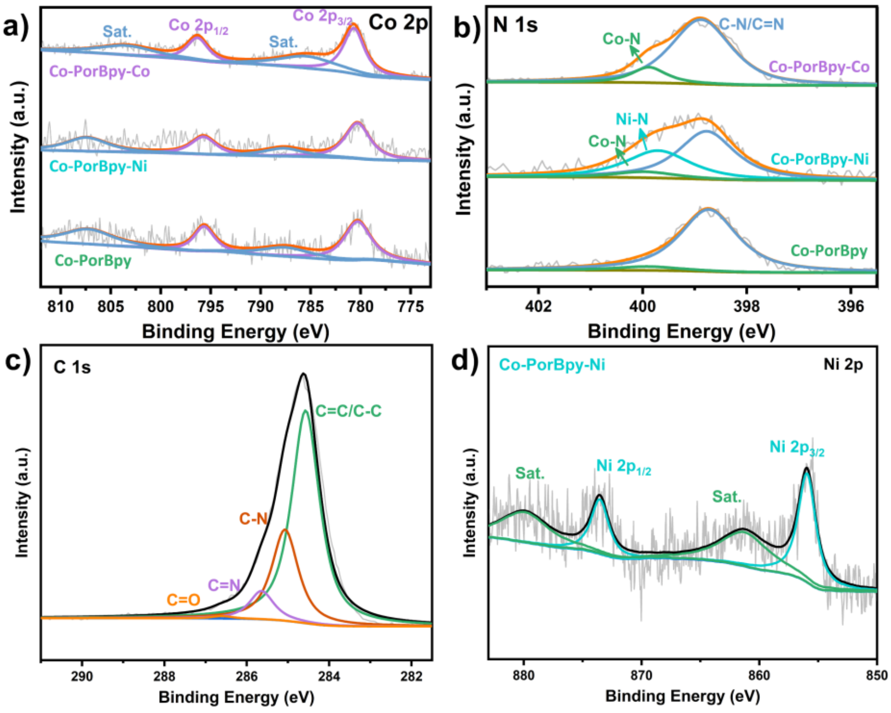

Figure S4. XPS Co 2p (a) and N 1s (b) spectra of Co-PorBpy, Co-PorBpy-Ni, and Co-PorBpy-Co, XPS C 1s (c) and Ni 2p (d) spectrum of Co-PorBpy-Ni.

Table S2. Element content measured by the corresponding XPS data

Element species	Co-PorBpy (at%)	Co-PorBpy-Co (at%)	Co-PorBpy-Ni (at%)
C	92.48	87.77	91.07
N	6.62	9.96	6.56
Co	0.90	2.27	1.11
Ni	-	-	1.26

4. TGA Test

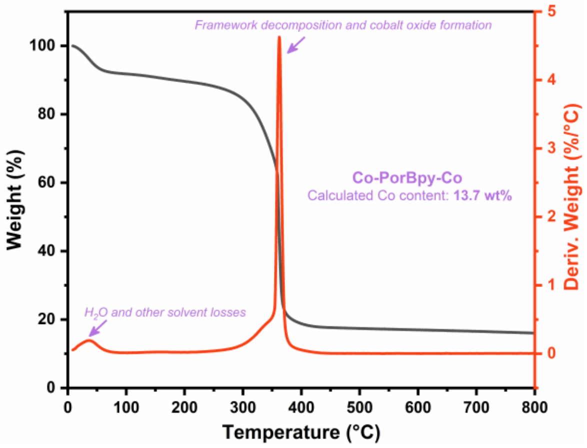

Figure S5. TGA of Co-PorBpy-Co.

5. SEM Images

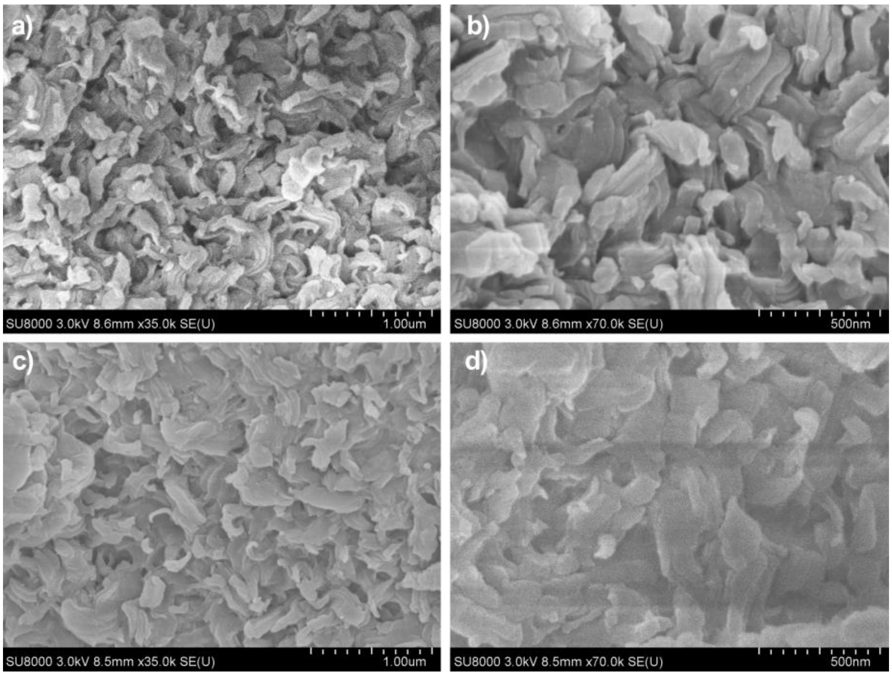

Figure S6. SEM images of (a, b) Co-PorBpy and (c, d) Co-PorBpy-Co.

6. COF Slabs for Band Structure Calculation

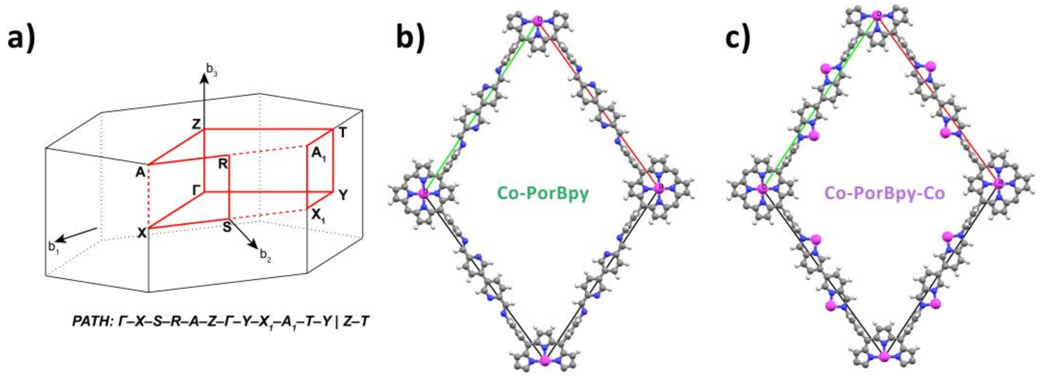

Figure S7. (a) Brillouin zone and corresponding K-path of ORCC lattice, (b) primitive cell of Co-PorBpy, (c) primitive cell of Co-PorBpy-Co.

7. PXRD Pattern

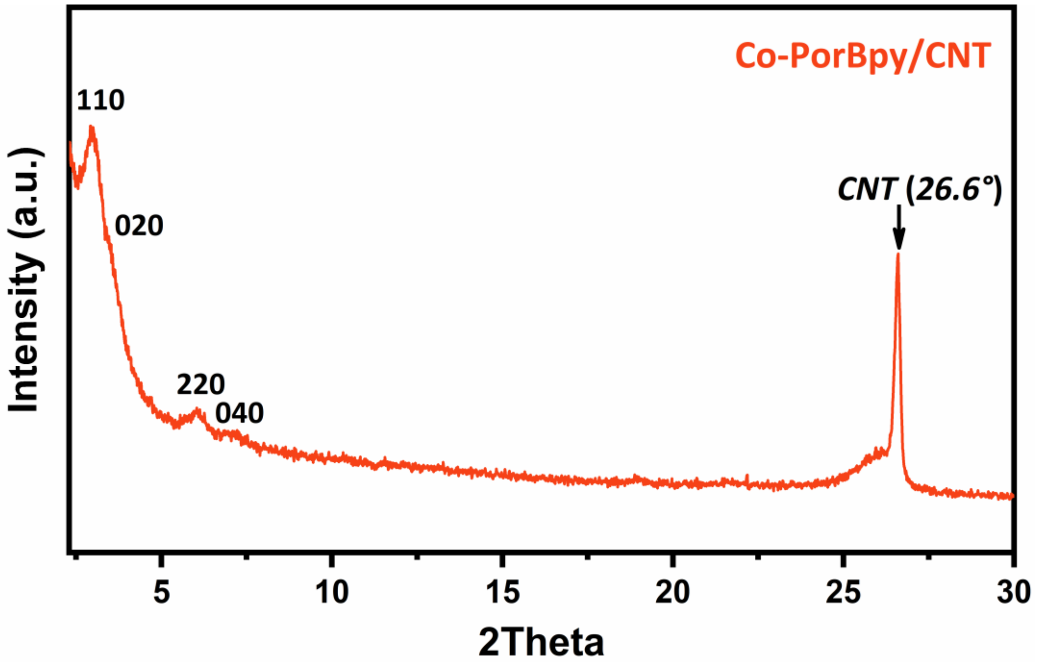

Figure S8. Experimental PXRD pattern of Co-PorBpy/CNT.

8. TEM Images

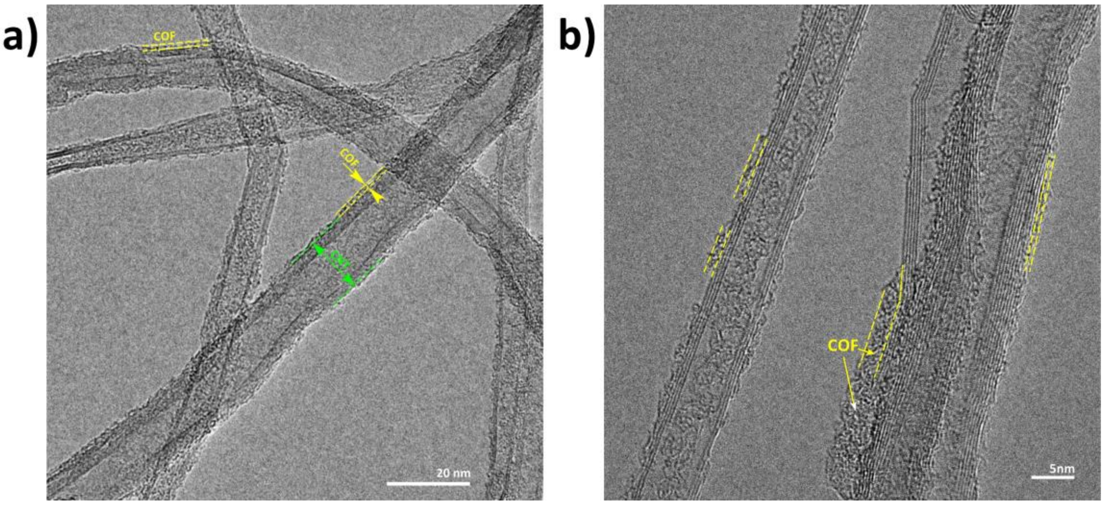

Figure S9. TEM images of Co-PorBpy/CNT.

9. ORR Tests

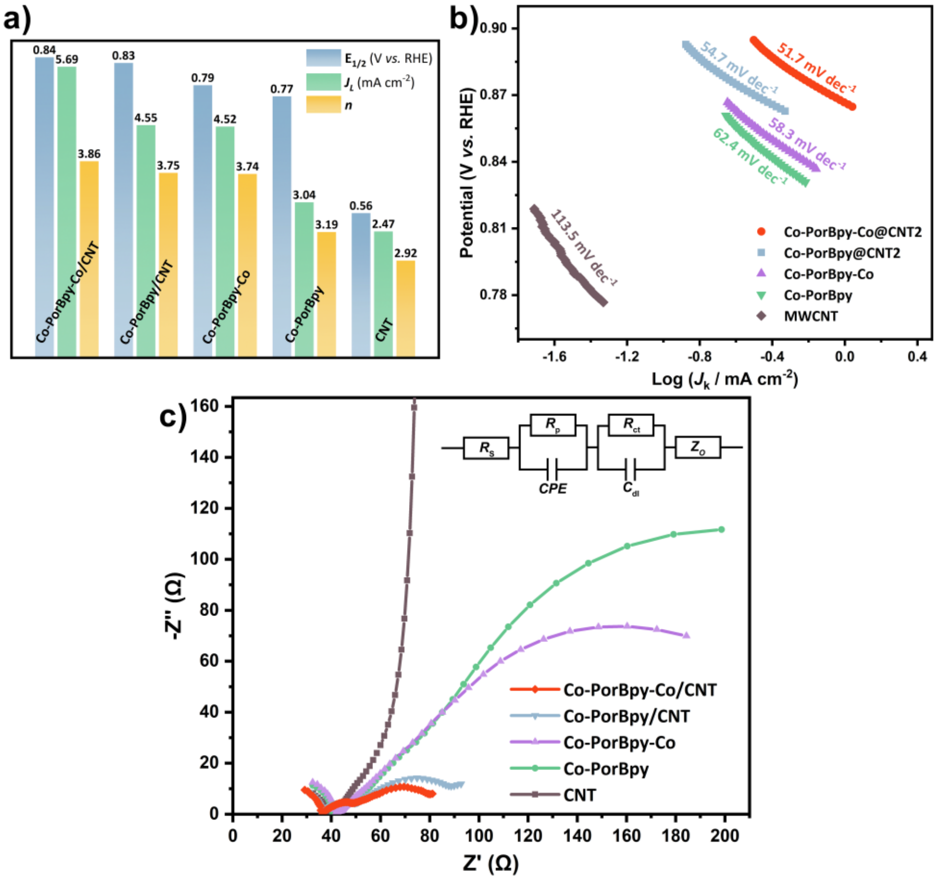

Figure S10. a) ORR activity comparison of the catalysts in this work, b) Tafel plots of the catalysts, c) EIS spectra of the catalysts (inset is the corresponding equivalent circuit).

10. Theoretical Computation Section

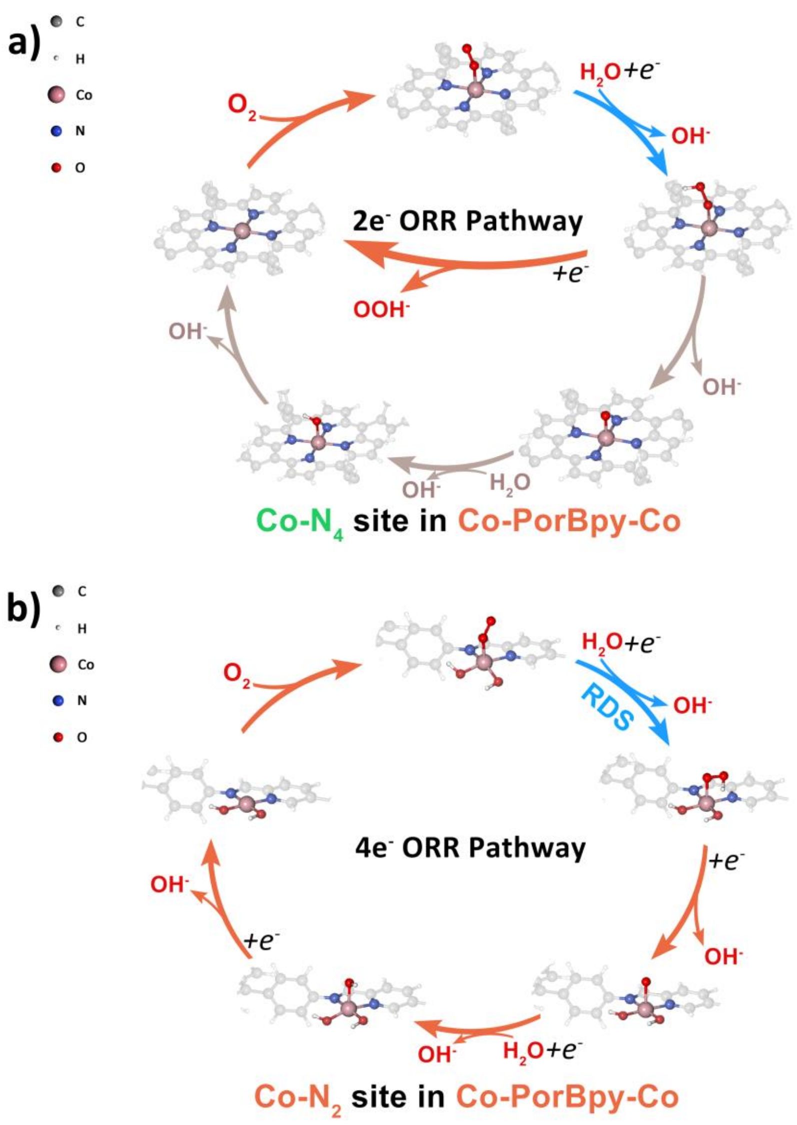

Figure S11. ORR pathway of (a) Co-N_4 site and (b) Co-N_2 site in **Co-PorBpy-Co**.

Table S3. DFT adsorption free energies of the ORR intermediates (O_2*, OOH*, O*, and OH*) for the active sites in **Co-PorBpy-Co** (U = 0 V). ORR limiting potentials and the rate-determine step (RDS) are displayed for each row.

Active Site	ΔG_{O2*} (eV)	ΔG_{OOH*} (eV)	ΔG_{O*} (eV)	ΔG_{OH*} (eV)	Limiting Barrier (eV)	Rate-determine Step
Co-N_2	4.137	3.298	1.879	0.873	0.391	O_2*→OOH*
Co-N_4	4.419	3.833	2.822	1.190	0.644	O_2*→OOH*

11. Electrocatalytic OER and Zn-air Battery Tests

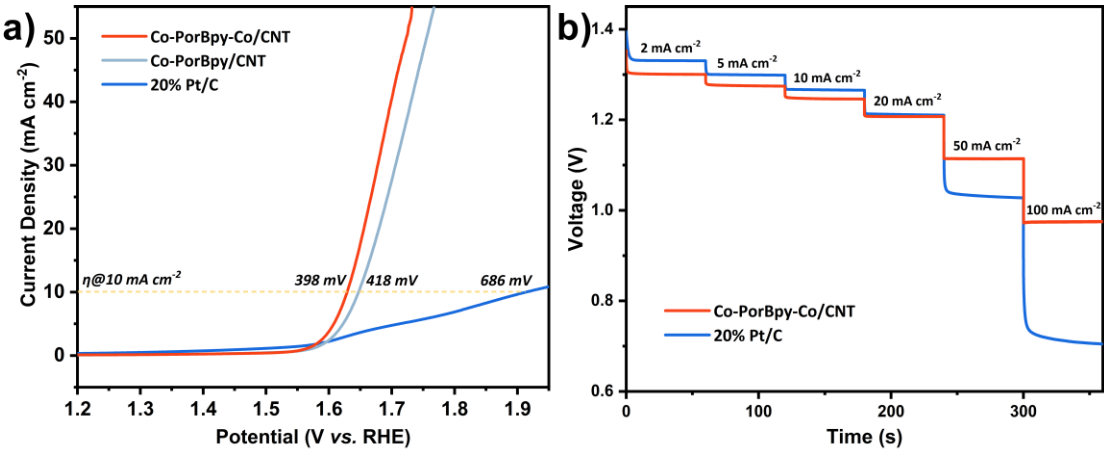

Figure S12. a) OER performance of Co-PorBpy/CNT, Co-PorBpy-Co/CNT, and 20 wt% Pt/C, b) galvanostatic discharge curves of the ZABs.

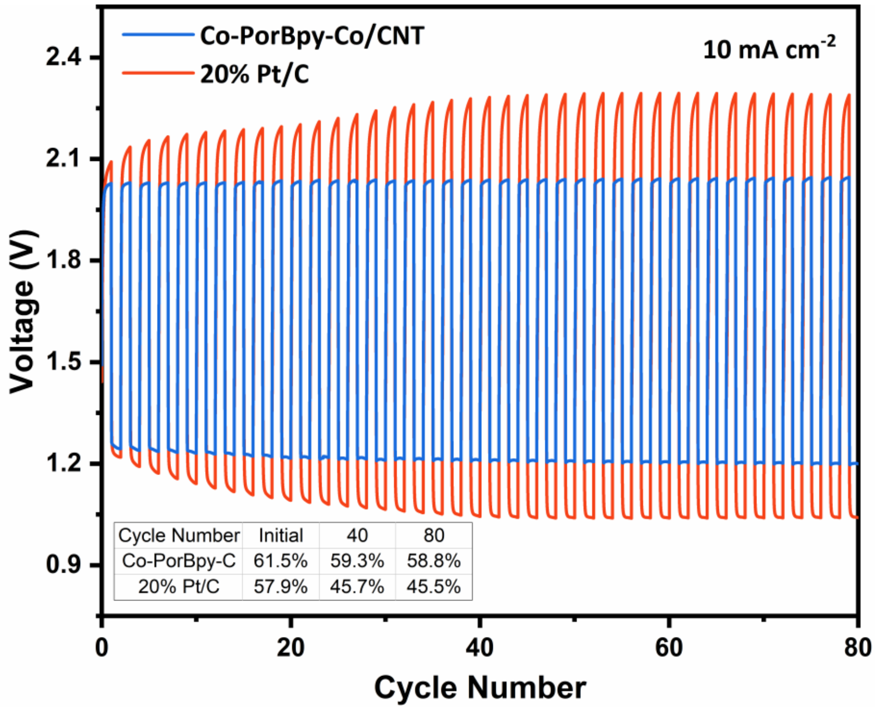

Cycle Number	Initial	40	80
Co-PorBpy-C	61.5%	59.3%	58.8%
20% Pt/C	57.9%	45.7%	45.5%

Figure S13. Cycling performance of the ZABs with Co-PorBpy-Co/CNT and 20 wt% Pt/C, the inset table displays the round-trip efficiencies of the ZABs.

12. Comparison Table

Table S4. Comparisons of the ORR activity of pristine COF catalysts.

Catalyst	Electrolyte	Half-wave Potential (V *vs.* RHE)	Current Density ($mA\ cm^{-2}$) @E (V *vs.* RHE)	Electron transfer number @E (V *vs.* RHE)	Rotate speed (rpm)	Ref.
**Co-PorBpy-Co/CNT**	**0.1M KOH**	**0.84**	**5.69@0.2**	**3.86@0.75**	**1600**	**This work**
NDI-CON	0.1M NaOH	~0.68	~3.72@0.3	3.6	1500	[9]
Co@TPA−PDI	0.1M KOH	~0.71	5.7@0.3	3.9	1600	[10]
PTM-CORF	0.1M KOH	~0.69	~5.7@0.17	3.89@0.6	1600	[11]
COF_{BTC}	0.1M KOH	0.91	~5.3@0.2	3.95@0.85	1600	[12]
CoCOF-Py-rGO	0.1M KOH	0.76	4.72@0.2	3.7@0.2	1600	[13]
COP/rGO	0.1M KOH	~0.72	~4.31@0.2	3.7@0.75	1600	[14]
TAPA-PG	0.1M KOH	~0.57	~4.16@0.2	3.84@0.3	1600	[15]
Co@TAPA-PG	0.1M KOH	~0.63	~4.8@0.2	3.63	1600	[15]
JUC-528	0.1M KOH	0.70	~5.0@0.2	3.81@0.2	1600	[16]
G@POF-Co	0.1M KOH	0.81	~5.43@0.2	3.8	1600	[17]
Co-TP-COF	0.1M KOH	0.73	4.8@0.3	3.7	1600	[18]
CC-3	0.1M KOH	0.828	~5.89@0.6	3.86	1600	[19]

All of these parameters with “~” are obtained by the graph of LSV curves at 1600 rpm.

13. Reference

[1] B. Q. Li, C. X. Zhao, S. Chen, J. N. Liu, X. Chen, L. Song, Q. Zhang, *Adv. Mater.* **2019**, e1900592.

[2] P. Giannozzi, S. Baroni, N. Bonini, M. Calandra, R. Car, C. Cavazzoni, D. Ceresoli, G. L. Chiarotti, M. Cococcioni, I. Dabo, A. Dal Corso, S. de Gironcoli, S. Fabris, G. Fratesi, R. Gebauer, U. Gerstmann, C. Gougoussis, A. Kokalj, M. Lazzeri, L. Martin-Samos, N. Marzari, F. Mauri, R. Mazzarello, S. Paolini, A. Pasquarello, L. Paulatto, C. Sbraccia, S. Scandolo, G. Sclauzero, A. P. Seitsonen, A. Smogunov, P. Umari, R. M. Wentzcovitch, *J. Phys.: Condens. Matter* **2009**, *21*, 395502.

[3] P. Giannozzi, O. Andreussi, T. Brumme, O. Bunau, M. Buongiorno Nardelli, M. Calandra, R. Car, C. Cavazzoni, D. Ceresoli, M. Cococcioni, N. Colonna, I. Carnimeo, A. Dal Corso, S. de Gironcoli, P. Delugas, R. A. DiStasio, A. Ferretti, A. Floris, G. Fratesi, G. Fugallo, R. Gebauer, U. Gerstmann, F. Giustino, T. Gorni, J. Jia, M. Kawamura, H. Y. Ko, A. Kokalj, E. Kucukbenli, M. Lazzeri, M. Marsili, N. Marzari, F. Mauri, N. L. Nguyen, H. V. Nguyen, A. Otero-de-la-Roza, L. Paulatto, S. Ponce, D. Rocca, R. Sabatini, B. Santra, M. Schlipf, A. P. Seitsonen, A. Smogunov, I. Timrov, T. Thonhauser, P. Umari, N. Vast, X. Wu, S. Baroni, *J. Phys.: Condens. Matter* **2017**, *29*, 465901.

[4] J. P. Perdew, K. Burke, M. Ernzerhof, *Phys. Rev. Lett.* **1996**, *77*, 3865-3868.

[5] P. E. Blochl, *Phys. Rev. B* **1994**, *50*, 17953-17979.

[6] A. D. Corso, *Comput. Mater. Sci.* **2014**, *95*, 337-350.

[7] A. Hjorth Larsen, J. Jorgen Mortensen, J. Blomqvist, I. E. Castelli, R. Christensen, M. Dulak, J. Friis, M. N. Groves, B. Hammer, C. Hargus, E. D. Hermes, P. C. Jennings, P. Bjerre Jensen, J. Kermode, J. R. Kitchin, E. Leonhard Kolsbjerg, J. Kubal, K. Kaasbjerg, S. Lysgaard, J. Bergmann Maronsson, T. Maxson, T. Olsen, L. Pastewka, A. Peterson, C. Rostgaard, J. Schiotz, O. Schutt, M. Strange, K. S. Thygesen, T. Vegge, L. Vilhelmsen, M. Walter, Z. Zeng, K. W. Jacobsen, *J. Phys.: Condens. Matter* **2017**, *29*, 273002.

[8] M. W. Chase, *NIST-JANAF Thermochemical Tables, 4th Edition*, American Institute of Physics, **1998**.

[9] S. Royuela, E. Martinez-Perinan, M. P. Arrieta, J. I. Martinez, M. M. Ramos, F. Zamora, E. Lorenzo, J. L. Segura, *Chem. Commun.* **2020**, *56*, 1267-1270.

[10] S. Bhattacharyya, D. Samanta, S. Roy, V. P. Haveri Radhakantha, T. K. Maji, *ACS Appl. Mater. Interfaces* **2019**, *11*, 5455-5461.

[11] S. Wu, M. Li, H. Phan, D. Wang, T. S. Herng, J. Ding, Z. Lu, J. Wu, *Angew. Chem. Int. Ed.* **2018**, *57*, 8007-8011.

[12] P. Peng, L. Shi, F. Huo, C. Mi, X. Wu, S. Zhang, Z. Xiang, *Sci. Adv.* **2019**, *5*, eaaw2322.

[13] Q. Zuo, G. Cheng, W. Luo, *Dalton Trans.* **2017**, *46*, 9344-9348.

[14] J. Guo, C. Y. Lin, Z. Xia, Z. Xiang, *Angew. Chem. Int. Ed.* **2018**, *57*, 12567-12572.

[15] A. Singh, D. Samanta, T. K. Maji, *ChemElectroChem* **2019**, *6*, 3756-3763.

[16] D. Li, C. Li, L. Zhang, H. Li, L. Zhu, D. Yang, Q. Fang, S. Qiu, X. Yao, *J. Am. Chem. Soc.* **2020**, *142*, 8104-8108.

[17] B. Q. Li, S. Y. Zhang, X. Chen, C. Y. Chen, Z. J. Xia, Q. Zhang, *Adv. Funct. Mater.* **2019**, *29*, 1901301.

[18] J. Y. Yue, Y. T. Wang, X. Wu, P. Yang, Y. Ma, X. H. Liu, B. Tang, *Chem. Commun.* **2021**, *57*, 12619-12622.

[19] C. Liu, F. Liu, H. Li, J. Chen, J. Fei, Z. Yu, Z. Yuan, C. Wang, H. Zheng, Z. Liu, M. Xu, G. Henkelman, L. Wei, Y. Chen, *ACS Nano* **2021**, *15*, 3309-3319.
